# Socioeconomic disparities in the uptake of breast and cervical cancer screening in Italy: a cross sectional study

**DOI:** 10.1186/1471-2458-12-99

**Published:** 2012-02-03

**Authors:** Gianfranco Damiani, Bruno Federico, Danila Basso, Alessandra Ronconi, Caterina Bianca Neve Aurora Bianchi, Gian Marco Anzellotti, Gabriella Nasi, Franco Sassi, Walter Ricciardi

**Affiliations:** 1 Department of Public Health, Università Cattolica del Sacro Cuore, Largo Francesco Vito 1, 00168 Rome, Italy; 2 Department of Health and Sport Sciences, Università di Cassino, Via S. Angelo snc, 03043 Cassino (FR), Italy; 3 Health Division, Directorate for Employment, Labour and Social Affairs, OECD-Organization for economic Cooperation and Development, 2 rue André Pascal, 75775 Paris, Cedex 16, France

## Abstract

**Background:**

Breast and cervical cancer screening are widely recognized as effective preventive procedures in reducing cancer mortality. The aim of this study was to evaluate the impact of socioeconomic disparities in the uptake of female screening in Italy, with a specific focus on different types of screening programs.

**Methods:**

A cross-sectional study was conducted using data from the 2004-2005 national health interview survey. A sample of 15, 486 women aged 50-69 years for mammography and one of 35, 349 women aged 25-64 years for Pap smear were analysed. Logistic regression models were used to estimate the association between socioeconomic factors and female screening utilization.

**Results:**

Education and occupation were positively associated with attendance to both screening. Women with higher levels of education were more likely to have a mammogram than those with a lower level (OR = 1.28; 95% CI = 1.10-1.49). Women of intermediate and high occupational classes were more likely to use breast cancer screening (OR = 1.77; 95% CI = 1.55-2.03, OR = 1.63; 95% CI = 1.40-1.91) compared to unemployed women. Women in the highest occupational class had a higher likelihood of cervical cancer screening compared to those in the lowest class (OR = 1.81; 95% CI = 1.63-2.01). Among women who attended screening, those with lower levels of education and lower occupational classes were more likely than more advantaged women to attend organized screening programs rather than being screened on the basis of their own initiative.

**Conclusions:**

Inequalities in the uptake of female screening widely exist in Italy. Organized screening programs may have an important role in increasing screening attendance and tackling inequalities.

## Background

Breast and cervical cancer have both high morbidity and mortality rates in Italy. In 2006, 36, 634 new cases of breast cancer were diagnosed and 11, 476 deaths were registered. In the same year 3, 418 new cases of cervical carcinoma were diagnosed, whereas 351 deaths due to cervical cancer and 2, 404 deaths due to cancer of the uterus not otherwise specified were recorded [1]. This disease burden can be reduced if cases are detected and treated early. Education helps people recognize early signs of cancer and seek prompt medical attention for symptoms, while screening programs, which include mammography for breast cancer, and Pap smear for cervical cancer, allow the early identification of cancer or pre-cancer before signs are recognizable [2].

Screening for breast and cervical cancer are strongly related with a reduction in cancer mortality [3]. Evidence-Based screening plans and European guidelines recommend a mammography every 2 years for women aged 50-69 and Pap test every 3 years for women aged 25-64 [4-6].

In Italy, in accordance to the National Health Plan, a National Plan for Prevention was developed to promote women's cancer screening. Cervical and breast cancer screening programs, promoted since the 90s, are developed at a regional level and offered free of charge to women of target age groups (25-64 for cervical cancer and 50-69 for breast cancer). Although participation rates generally increased in recent years, they were substantially lower than those recommended by international guidelines. In 2008, participation in organized screening programs was 40% and 55% for cervical cancer and breast cancer, respectively [6]. These figures are substantially lower than those set by the European guidelines of 85% and 70%, respectively [7].

Socioeconomic factors were shown to be strongly related to the use of preventive services [8-11]. Disparities in the utilization of female screening were widely identified [11-13]. Comparative studies on the use of preventive services in Europe showed inequalities in the participation to screening programs, although the size of the inequality varied among countries [14,15]. Women with lower health literacy are less likely to carry out routine cancer screening [16,17]. Ethnic minority, old age and low socioeconomic status are all accompanied by a low chance of undergoing cancer screening procedures [18].

In the US characteristics associated with lower rates of Pap test use included low family income and low educational attainment [19]; income and educational level were positively associated with mammography practice in a French population-based study [20].

Recent Italian studies generally focused on the effectiveness of screening programs [21,22]. Other Italian studies reported significant regional and educational inequalities [17,23].

This study aims to assess the association between socioeconomic status and the use of female cancer screening. A further objective was to evaluate whether socioeconomic factors are associated with adherence to organised screening programs versus opportunistic ones.

## Methods

A cross-sectional study was conducted using data from the National Survey on "Health conditions and health care services use", a five-yearly nationwide survey conducted by the Italian National Centre for Statistics (Istat) in December 2004-March 2005. Data were provided in an anonymised electronic dataset which is publicly available at Istat website [24]. Information was collected through face to face interviews and self-administered questionnaires. This analysis focused on women without self-reported history of cancer.

According to cancer screening guidelines the analysis was conducted on a subgroup of women aged 25-64 years for Pap smear and on a subgroup of women aged 50-69 years for mammography.

The survey contained the following questions: "Have you ever had a mammography/Pap test without having symptoms?"; "In case you had at least one mammography/Pap test, did you have other tests afterwards?"; "In case you had at least one mammography/Pap test, how often did you have the following tests afterwards?".

The frequency of screening practices was considered "appropriate" if women reported having a mammogram every 2 years and a Pap test every 3 years according to European guidelines [5,6]. For women aged 50-53 we considered as regular prevention having only one mammography, whereas for women aged 25-29 we considered as regular prevention having only one Pap test. With regards to the comparison between organized and opportunistic screening, we defined a dichotomous variable as whether or not a women reported having had Pap test or mammogram on invite of National Health Program at the most recent screening. This second analysis was performed on the subset of women who reported having an appropriate frequency of screening.

Demographic variables, such as age (from 50 to 69 for breast cancer screening and from 25 to 64 for cervical cancer screening), region of residence and marital status were considered as independent variables. Other independent variables considered were Body Mass Index (BMI), smoking status and self-assessed health status, because they were all found to be correlated to the use of female screening [25,26].

Educational level (primary school or less, secondary school, and high school and over) and occupational class (with the categories high, intermediate, low and non-working) were used as indicators of socioeconomic status. The non-working category included students, those unable to work, those in search of occupation, retired and housewives. In the breast cancer sample housewives were 86.5% of the "non-working" category, while in the cervical cancer sample they were 70.8% of the same category. Occupational class was classified according to the UK National Statistics Socio-economic Classification (NS-SEC) [27]. NS-SEC class was derived using the full derivation method based on the 1990 Standard Operational Classification (SOC) codes combining data on occupation and employment status (whether an employer, self-employed or employee; whether a supervisor, manager).

Descriptive statistics were used to describe the study population. Separate multivariate logistic regression models were developed to examine the relationship between all explanatory variables and the outcomes of interest. For each final model, adjusted odds ratios (OR) and their 95% confidence intervals (CI) were calculated. Sampling weights in all analyses were used, in order to reflect the multistage sampling design of the survey.

## Results

### Breast cancer screening

Table 1 describes the characteristics of the sample (N = 15, 486). About 43.4% of women lived in Northern Italy and 11, 310 women (73.0%) were married. About 50% of the sample reported a "fair" health status and only 8.5% a "bad" or "very bad" status. Approximately half had less than secondary school education and 38.2% was in the low occupational class. Women who underwent routine breast cancer screening were 47.0% of the sample. Table 2 shows prevalence rates of having regular screening by sample characteristics. Women with the highest level of education attended more frequently breast cancer screening than women with the lowest educational level (57.0% vs. 40.5%). There was a strong positive association between occupational class and attendance to breast cancer screening.

**Table 1:** Characteristics of the sample*

	*Breast cancer screening*		*Cervical cancer screening*	
	(N = 15, 486)		(N = 35, 349)	
	N	Proportion (%)	N	Proportion (%)
***Age groups***				
25-34			8746	24.7
35-44			10201	28.9
45-54			8677	24.5
55-64			7725	21.9

50-54	4184	27.0		
55-59	4299	27.8		
60-64	3426	22.1		
65-69	3577	23.1		
***Region of residence***				
North-western Italy	3599	23.2	12383	35.0
North-eastern Italy	3127	20.2	2355	21.5
Central-Italy	2833	18.3	6241	20.2
Southern Italy	4213	27.2	10405	29.4
Italian Islands	1714	11.1	3965	11.2
***Marital status***				
Single	1067	6.9	7257	20.5
Married	11310	73.0	23782	67.3
Separated/Divorced	914	5.9	2723	7.7
Widowed	2195	14.2	1587	4.5
***Body mass index***				
Normal weight	7564	48.8	22342	63.2
Underweight	305	2.0	1889	5.3
Overweight	5378	34.7	8128	23
Obese	2239	14.5	2990	8.5
***Self-assessed health status***				
Good/Very good	6414	41.4	22771	64.4
Fair	7751	50.1	11310	32.0
Bad/Very bad	1321	8.5	1268	3.6
***Cigarette smoking status***				
Current	2216	14.3	6474	18.3
Former	2565	16.6	5869	16.6
Never	10705	69.1	23006	65.1
***Level of education***				
Primary school or less	7603	49.1	7082	20.1
Secondary school	3900	25.2	11214	31.7
High school and over	3983	25.7	17053	48.2
***Occupational class***				
Non-working	4941	31.9	9806	27.8
Low	5915	38.2	11643	32.9
Intermediate	3167	20.5	10223	28.9
High	1463	9.4	3677	10.4
***Mammography***				
One every two years	7274	47.0		
Less than two or none	8212	53.0		
***Pap test***				
One every three years			18417	52.1
Less than three or none			16932	47.9

*Italian National Health Interview Survey, 2004-2005 [24].

**Table 2:** Prevalence rates of having regular mammography and Pap test by sample characteristics

	*Women that performed a **mammography **every two years*		*Women that performed a Pap test every three years*	
	*n*	*%*	*n*	*%*
***Age groups***				
25-34			3718	42.5
35-44			5482	53.7
45-54			5084	58.6
55-64			4133	53.5

50-54	2409	57.4		
55-59	2038	47.6		
60-64	1478	43.9		
65-69	1349	38.1		
***Region of residence***				
North-western Italy	1985	56.4	4795	63.1
North-eastern Italy	1959	62.7	5236	73.3
Central-Italy	1507	50.3	3753	60.1
Southern Italy	1308	29.3	3403	32.7
Italian Islands	515	28.1	1230	31.0
***Marital status***				
Single	402	38.4	2739	37.7
Married	5608	49.8	13376	56.2
Separated/Divorced	419	46.1	1529	56.2
Widowed	845	38.2	773	48.7
***Body mass index***				
Normal weight	3732	49.8	11981	53.6
Underweight	156	50.0	945	50.0
Overweight	2451	45.7	4120	50.7
Obese	935	41.1	1371	45.9
***Self-assessed health status***				
Good/Very good	3087	48.7	11540	50.7
Fair	3690	47.5	6279	55.5
Bad/Very bad	497	37.5	598	47.2
***Cigarette smoking status***				
Current	1130	44.7	3452	49.0
Former	1407	54.8	3690	63.5
Never	4737	50.2	11275	53.9
***Level of education***				
Primary school or less	3052	40.5	3114	44.0
Secondary school	1951	49.5	5632	50.5
High school and over	2271	57.0	9671	56.7
***Occupational class***				
Non-working	1800	35.4	3598	63.3
Low	2788	47.9	6142	52.8
Intermediate	1863	59.5	6371	62.3
High	823	57.5	2306	62.7

Results of logistic regression models are shown in Table 3. Age, region of residence, marital status, education and social class were significant predictors of regular breast cancer screening after adjusting for the other covariates. Women of 55 years and older were less likely to have had a mammogram within the clinically recommended 2 year-time period. The likelihood to perform breast cancer screening was lower in central (OR = 0.83; 95% CI = 0.73-0.96) and southern (OR = 0.30; 95% CI = 0.26-0.34) than in northern regions. Married women resulted more likely to have regular prevention (OR = 1.83; 95% CI = 1.56-2.15). Women with a higher level of education were more likely to have a mammogram within the past 2 years than those with a lower level (OR = 1.28; 95% CI = 1.10-1.49). Women of intermediate and high occupational classes were more likely to use screening (OR = 1.77; 95% CI = 1.55-2.03, OR = 1.63; 95% CI = 1.40-1.91) compared to the unemployed ones. Obese women had a lower likelihood to have a mammogram than normal weight ones (OR = 0.87; 95% CI = 0.77-0.98). Significant interactions were found between the highest educated and living in central and Southern Italy: as a result, educational inequalities were largest in Southern Italy and lowest in Central Italy.

**Table 3:** Odds ratio of having regular prevention by mammography

	*ORs (CI 95%)*	*Linearized standard errors*	*p-value*
*Age groups*			
50-54	1		
55-59	0.64 (0.57-0.71)	0.04	< 0.001
60-64	0.58 (0.51-0.65)	0.03	< 0.001
65-69	0.47 (0.42-0.53)	0.03	< 0.001
*Region of residence*			
North-western Italy	1		
North-eastern Italy	1.30 (1.16-1.46)	0.08	< 0.001
Central-Italy	0.83 (0.73-0.96)	0.06	< 0.05
Southern Italy	0.30 (0.26-0.34)	0.02	< 0.001
Italian Islands	0.32 (0.27-0.37)	0.02	< 0.001
***Marital status***			
Single	1		
Married	1.83 (1.56-2.15)	0.15	< 0.001
Separated/Divorced	1.15 (0.93-1.43)	0.13	0.206
Widowed	1.32 (1.10-1.60)	0.13	< 0.05
***Body mass index***			
Normal weight	1		
Underweight	0.88 (0.64-1.20)	0.14	0.403
Overweight	1.04 (0.95-1.13)	0.05	0.405
Obese	0.87 (0.77-0.98)	0.05	< 0.05
***Self-assessed health status***			
Good/Very good	1		
Fair	1.17 (1.07-1.27)	0.05	< 0.001
Bad/Very bad	1.01 (0.86-1.17)	0.08	0.957
***Cigarette smoking status***			
Never	1		
Former	1.11 (0.99-1.24)	0.06	0.084
Current	0.95 (0.85-1.06)	0.05	0.361
***Level of education***			
Primary school or less	1		
Secondary school	1.08 (0.97-1.19)	0.05	0.147
High school and over	1.28 (1.10-1.49)	0.10	< 0.001
***Occupational class***			
Non-working	1		
Low	1.23 (1.12-1.36)	0.06	< 0.001
Intermediate	1.77 (1.55-2.03)	0.12	< 0.001
High	1.63 (1.40-1.91)	0.13	< 0.001
***Interaction between Region of residence and Level of education ***			
North-western Italy*Primary school or less	1		
Central-Italy*High school and over	0.77 (0.61-0.98)	0.09	< 0.05
Southern Italy*High school and over	1.38 (1.13-1.70)	0.15	< 0.001

**Figure 1:**
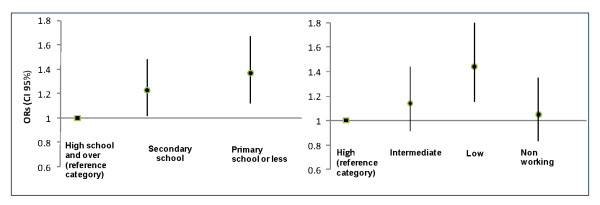
**Odds Ratio of attending an organized mammography screening program versus opportunistic screening by level of education and social class adjusted for age, regional residence, marital status, BMI, smoking status and self-assessed status**.

Figure 1 shows the ORs of attending an organized mammography screening program versus opportunistic screening by level of education and social class adjusting for age, regional residence, marital status, BMI, smoking status and self-assessed health status. The figure shows that women with lower educational level had a higher likelihood of attending an organized mammography screening program than more educated women (OR = 1.37; 95% CI = 1.12-1.67). In addition, low social class was associated with a greater use of organized screening program (OR = 1.44; 95% CI = 1.15-1.81) compared to a higher class.

### Cervical cancer screening

Table 1 summarizes the characteristics of the 35, 349 women included in the study. About half of the sample had a high educational level (48.2%), only 10.4% were in the high occupational class, while there were not considerable differences in the percentages of non-working (27.8%), low (32.9%) and intermediate class (28.9%). More than 50% of women had a Pap test within the past 3 years.

A positive educational gradient was found for attendance to cervical cancer screening (Table 2). Attendance ranged from 56.7% among the highest educated to 44.0% among the lowest educated. Women in the low occupational class showed a lower attendance rate compared to the other occupational groups.

Results of logistic regression models are shown in Table 4. Women aged 35-64 years were more likely to undergo screening than women aged 25-34 years. A regional gradient was found in the regular uptake of cervical cancer screening: women living in Southern Italy and main islands, used Pap test less frequently than women living in North Italy with an OR of 0.38 (95% CI = 0.33-0.43) and an OR of 0.30 (95% CI = 0.27-0.34), respectively. Married women had a higher likelihood to have a Pap test (OR = 2.41; 95% CI = 2.23-2.60) than single women. Obese women reported a lower likelihood of cancer screening (OR = 0.77; 95% CI = 0.70-0.85) than normal weight ones. Former smoking status was an important predictor of regular Pap test attendance (OR = 1.36; 95% CI = 1.25-1.47). Women with a high socioeconomic status had a higher likelihood of cervical cancer screening compared to those with a lower status. The ORs were 1.91 (95% CI = 1.72-2.13) for women with a high school level or a higher level and 1.81 (95% CI = 1.63-2.01) for those with a high occupational class. The significant interaction terms in this model imply that higher levels of education in the South are less strongly associated with cervical cancer screening than in the rest of Italy.

**Table 4:** Odds ratio of having regular prevention by Pap test

	*ORs(CI 95%)*	*Linearized standard errors*	*p-value*
*Age groups*			
25-34	1		
35-44	1.21 (1.12-1.31)	0.05	< 0.001
45-54	1.62 (1.49-1.77)	0.07	< 0.001
55-64	1.45 (1.32-1.60)	0.07	< 0.001
*Region of residence*			
North-western Italy	1		
North-eastern Italy	1.65 (1.52-1.80)	0.07	< 0.001
Central-Italy	0.87 (0.80-0.95)	0.04	< 0.05
Southern Italy	0.38 (0.33-0.43)	0.03	< 0.001
Italian Islands	0.30 (0.27-0.34)	0.02	< 0.001
***Marital status***			
Single	1		
Married	2.41 (2.23-2.60)	0.09	< 0.001
Separated/Divorced	1.69 (1.50-1.90)	0.10	< 0.001
Widowed	1.78 (1.54-2.07)	0.14	< 0.001
***Body mass index***			
Normal weight	1		
Underweight	0.98 (0.87-1.12)	0.04	0.790
Overweight	0.90 (0.84-0.97)	0.08	< 0.05
Obese	0.77 (0.70-0.85)	0.06	< 0.001
***Self-assessed health status***			
Good/Very good	1		
Fair	1.22 (1.14-1.29)	0.04	< 0.001
Bad/Very bad	1.10 (0.95-1.28)	0.07	0.206
***Cigarette smoking status***			
Never	1		
Former	1.36 (1.25-1.47)	0.10	< 0.001
Current	1.10 (1.03-1.18)	0.05	< 0.05
***Level of education***			
Primary school or less	1		
Secondary school	1.44 (1.31-1.59)	0.05	< 0.001
High school and over	1.91 (1.72-2.13)	0.10	< 0.001
***Occupational class***			
Non-working	1		
Low	1.28 (1.19-1.38)	0.05	< 0.001
Intermediate	1.73 (1.60-1.89)	0.07	< 0.001
High	1.81 (1.63-2.01)	0.10	< 0.001
***Interaction between Region of residence and Level of education***			
North-western Italy*Primary school or less	1		
Southern-Italy*Secondary school	0.78 (0.66-0.91)	0.06	< 0.05
Southern Italy*High school and over	0.83 (0.72-0.96)	0.06	< 0.05

**Figure 2:**
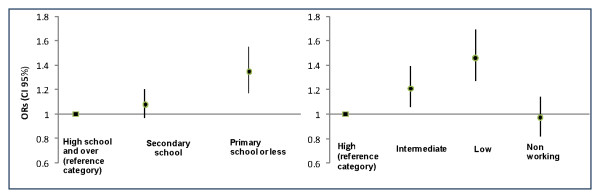
**Odds Ratio of attending an organized cervical cancer screening program versus opportunistic screening by level of education and social class adjusted for age, regional residence marital status, BMI, smoking status and self-assessed status**.

Figure 2 shows the OR of attending an organized cervical cancer screening program versus opportunistic screening by level of education and social class adjusting for age, regional residence, marital status BMI, smoking status and self-assessed health status. The lowest educated women had a higher likelihood of using organized screening than those with a higher educational level (OR = 1.35; 95% CI = 1.17-1.55). Women of lower occupational class had a higher odds of organized screening program attendance compared with women in the highest occupational class (OR = 1.46; 95% CI = 1.27-1.69).

## Discussion

In our study, we investigated inequity in breast and cervical cancer screening among Italian women. Our study suggests the presence of important inequalities in the use of these preventive services: both lower level of education and occupational class are strongly associated with underutilization of screening, despite coverage of most expenses for such preventive services by the Italian National Health System. However, among women who attended screening, those with lower level of education and lower occupational class were more likely to attend organized screening program rather than being screened on the basis of their own initiative.

These findings are consistent with the results of other international studies [8,13,28-30] reporting that women with lower socioeconomic status are less likely to undergo cancer screening. Sabates and Feinstein investigated the role of education in the uptake of cervical cancer screening in Britain; they found that continuing adult learning has a direct impact on the uptake of preventative screening which is not reduced by income, occupation or social class [31]. Furthermore, Rakowski et al. highlighted the positive influence of education on preventive behaviours [32]. Recently, a study on the use of breast and cervical cancer screening among European countries found that inequalities existed in some countries and were related to the type of screening program [14]. Contextual effects may also be important: it was shown that less educated women living in metropolitan areas with a lower proportion of low-education residents are less likely to undergo cancer screening, compared to women with similar level of education in other metropolitan areas. This may be due to socioeconomic factors or to the lack of culturally appropriate and accessible preventive health care services in the areas in which women live [16]. On the other hand, Achat et al. in 2005 demonstrated the existence of a weak association between socioeconomic status and regularity of mammography among Australian women when preventive programs were available without direct charge [33].

Referring to the relationship between socioeconomic status and adherence to organized screening programs versus opportunistic screening, our results are in line with several studies showing that women who attended an organized breast cancer screening program were more likely to be of a lower socioeconomic status [20,34]. These studies suggested that screening programs appeared to attract disadvantaged women who did not usually undergo screening. Similarly, a national study reported lower participation in organized screening program in more educated women, which was thought to reflect the greater extent of private purchase of screening outside public services [35]. In their study on the influence of type of screening program on the extent of inequality in some European countries, Palencia et al. reported large inequalities in countries without population-based cancer screening programs [14].

In contrast, other studies reported that organized screening programs assure a generic positive effect on coverage without clearly reducing the social gap [15,30,36,37]. More recently, results from two studies seemed to confirm that it is necessary to give programs longer periods of time since their start in order to observe any impact on inequalities [38,39].

In our study, we found that the association between socioeconomic status and mammography uptake was stronger for occupational status than for education. Women who do non-working were the most disadvantaged. This finding is similar to the one reported by Zackrisson et al. [40].

Our results show a positive association between female screening and marriage condition similar to other studies [33,41]. Being unmarried was a stronger predictor for not undergoing screening especially for Pap test. It may be that Pap test was often offered to married women as part of pre or post natal services [28]. In addition, according to Zackrisson et al. marital status could be considered as a proxy for social support [40].

Age was positively correlated to the uptake of Pap test whereas it was negatively correlated with the use of mammography. Conflicting findings are reported in the literature in this regard [42-44]. Higher rates found among older women for Pap smear may be due to a lower attention paid to preventive issues among younger generations.

We also considered the influence of BMI and smoking status on the use of preventive services. The role of obesity as a barrier to screening is a fairly recent research topic. This study reveals, as also shown by Datta [45], that women with BMI > 30 had a greater likelihood of non attending screening than women with normal weight. Cohen et al. discussed possible reasons for this association that were not necessarily weight-related, including embarrassment, discomfort and emotional barriers [46]. Results from a meta-analysis showed that obesity was inversely associated with the likelihood of having recently undergone a mammography [25].

In contrast with previous studies, our findings show that cigarettes smokers are not less likely that non smokers to use cancer preventive services [26,29]. Recently Ortiz et all found similar results, reporting that Pap screening was not associated with smoking status and other unhealthy behaviors [47]. We showed that former smokers tended to have higher attendance to screening than current smokers and people who were never smokers. This may be because former smokers decided to adopt a healthier lifestyle altogether. Similar results are reported by Rakowski et al. [48].

Despite the existence of free cancer prevention programs, the overall proportion of women that undertake regular screening tests is relatively small. Only half of the investigated women have had regular prevention, even though in Italy female screening programs have been existing for more than 10 years.

Deficit in utilization may be due to a lack of trust in the National Health Service and in its initiatives, as a consequence of the wide geographical heterogeneity in implementation of regional programs. Other reasons associated with poor adherence to screening may be the low perception in cancer screening efficacy, the fear of radiation mammography, the anxiety for the result and the fear of cancer.

In order to increase screening uptake rates, Duport et al. suggested that media campaigns should target women who were never screened or not regularly screened, underlying the importance of early diagnosis of breast cancer and the fact that screening is free of charge. On the other hand, benefits in terms of quality of organization about screening programs should be shown to women who underwent opportunistic mammography [20].

Our findings are subject to some limitations. First, there may be an effect of recall bias on self reported information about cancer screening practices: patients frequently tend to over-report their use of Pap test or mammogram and underreport the time lapse since their last screening [43,49].

Furthermore, several studies found that women's self-reported information varies according to the type of health care providers and to socio-demographic factors [50]. Secondly, useful information on some variables was not included in the survey questionnaire such as number of partners and parity.

A major strength of this study is that data were collected on a large national population-based sample. Furthermore, this sample provided detailed information about health status, socio-demographic characteristics and unhealthy behaviours.

In Italy, the 1998-2000 National Health Plan recommended that cancer screening programs should be introduced in every region [51]. Since 2005 the National Screening Observatory and the National Centre for Disease Control and Prevention have been working in partnership in order to control and support Regions in implementing screening programs.

Identifying reasons for failures of cancer screening is an important public health issue. In order to increase the proportion of women who carry out regular prevention it could be useful to improve the organization of screening services, for example through more flexible hours to meet the needs of women. Furthermore, it is important to involve the primary health sector to enhance and promote the spread of information on the benefits of screening to improve access to health services by increasing women compliance. Knowledge about socioeconomic status is essential for providing equal access to preventive care. Specific interventions at the national, regional and local level have to be designed in order to reduce disparities in screening utilisation by focusing on disadvantaged women. The implementation of organized screening programs may have an important role in increasing screening attendance and tackling socioeconomic inequalities.

## Conclusions

Inequalities in the uptake of female screening widely exist in Italy. Organized screening programs may have an important role in increasing screening attendance and tackling inequalities.

## Competing interests

The authors declare that they have no competing interests.

## Authors' contributions

GD, FS and WR contributed to the conception of this paper; GD, BF and DB designed the study. DB, CBNAB, GMA and GN selected articles that met the inclusion criteria and extracted data. AR conducted the statistical analysis.

All authors made substantial contributions to the interpretation of results and have read and approved the final version.

## Pre-publication history

The pre-publication history for this paper can be accessed here:

http://www.biomedcentral.com/1471-2458/12/99/prepub
